# Impact of Intermittent Presumptive Treatment for Malaria in Pregnancy on Hospital Birth Outcomes on the Kenyan Coast

**DOI:** 10.1093/cid/ciac509

**Published:** 2022-06-22

**Authors:** Alice Kamau, Moses Musau, Stella Mwakio, David Amadi, Amek Nyaguara, Philip Bejon, Anna C Seale, James A Berkley, Robert W Snow

**Affiliations:** Public Health Research, Kenya Medical Research Institute–Wellcome Trust Research Programme, Nairobi, Kenya; Public Health Research, Kenya Medical Research Institute–Wellcome Trust Research Programme, Nairobi, Kenya; Epidemiology and Demography, Kenya Medical Research Institute–Wellcome Trust Research Programme, Kilifi, Kenya; Epidemiology and Demography, Kenya Medical Research Institute–Wellcome Trust Research Programme, Kilifi, Kenya; Epidemiology and Demography, Kenya Medical Research Institute–Wellcome Trust Research Programme, Kilifi, Kenya; Epidemiology and Demography, Kenya Medical Research Institute–Wellcome Trust Research Programme, Kilifi, Kenya; Centre for Tropical Medicine and Global Health, Nuffield Department of Clinical Medicine, University of Oxford, Oxford, United Kingdom; Epidemiology and Demography, Kenya Medical Research Institute–Wellcome Trust Research Programme, Kilifi, Kenya; Epidemiology and Population Health, London School of Hygiene & Tropical Medicine, London, United Kingdom; College of Health and Medical Sciences, Haramaya University, Harar, Ethiopia; Warwick Medical School, University of Warwick, Coventry, United Kingdom; Public Health Research, Kenya Medical Research Institute–Wellcome Trust Research Programme, Nairobi, Kenya; Centre for Clinical Vaccinology and Tropical Medicine, Churchill Hospital, University of Oxford, Oxford, United Kingdom; Public Health Research, Kenya Medical Research Institute–Wellcome Trust Research Programme, Nairobi, Kenya; Centre for Tropical Medicine and Global Health, Nuffield Department of Clinical Medicine, University of Oxford, Oxford, United Kingdom

**Keywords:** IPTp-SP, SP resistance, low birthweight, stillbirths, Kenya

## Abstract

**Background:**

Intermittent preventive treatment (IPTp) for pregnant women with sulfadoxine–pyrimethamine (SP) is widely implemented for the prevention of malaria in pregnancy and adverse birth outcomes. The efficacy of SP is declining, and there are concerns that IPTp may have reduced impact in areas of high resistance. We sought to determine the protection afforded by SP as part of IPTp against adverse birth outcomes in an area with high levels of SP resistance on the Kenyan coast.

**Methods:**

A secondary analysis of surveillance data on deliveries at the Kilifi County Hospital between 2015 and 2021 was undertaken in an area of low malaria transmission and high parasite mutations associated with SP resistance. A multivariable logistic regression model was developed to estimate the effect of SP doses on the risk of low birthweight (LBW) deliveries and stillbirths.

**Results:**

Among 27 786 deliveries, 3 or more doses of IPTp-SP were associated with a 27% reduction in the risk of LBW (adjusted odds ratio [aOR], 0.73; 95% confidence interval [CI], .64–.83; *P* < .001) compared with no dose. A dose-response association was observed with increasing doses of SP from the second trimester linked to increasing protection against LBW deliveries. Three or more doses of IPTp-SP were also associated with a 21% reduction in stillbirth deliveries (aOR, 0.79; 95% CI, .65–.97; *P* = .044) compared with women who did not take any dose of IPTp-SP.

**Conclusions:**

The continued significant association of SP on LBW deliveries suggests that the intervention may have a non-malaria impact on pregnancy outcomes.

## Teaser

Results presented here suggest that intermittent preventive treatment for pregnant women with sulfadoxine–pyrimethamine (SP) remains associated with a reduced risk of low birthweight deliveries and stillbirths in an area with high levels of SP resistance on the Kenyan coast.

Malaria infection during pregnancy poses a risk to mothers and their fetuses. Reviews of the potential impact of *Plasmodium falciparum* infection in sub-Saharan Africa on maternal health and birth outcomes have highlighted the role played by subclinical infection on maternal anemia, placental infections, and low birthweight (LBW) deliveries [1–9]. Trials of 2 presumptive doses of sulfadoxine–pyrimethamine (SP) in the second and third trimesters between 1998 and 2008 showed significant reductions in maternal anemia and LBW [10, 11], notably in first and second pregnancies. SP is safe during second and third trimesters and operationally feasible when integrated into routine antenatal care (ANC) [12]. In 2004, the World Health Organization issued a policy recommendation that 2 doses of SP should be administered during ANC visits from the second trimester [13]. In 2012, this policy was revised to indicate that, “in areas of moderate-to-high malaria transmission, IPTp with SP is recommended for all pregnant women at each scheduled antenatal care visit; the first IPTp-SP dose should be administered as early as possible during the 2nd trimester; each SP dose should be given at least 1 month apart; and that the last dose of IPTp with SP can be administered up to the time of delivery, without safety concerns” [14].

Reducing the incidence of LBW, that is, newborns weighing less than 2500 g [15], is an important public health ambition given its association with premature mortality in infancy [16–18]. In 2010, modeled estimates suggested that there were between 0.5 and 1.2 million LBW deliveries attributed to malaria in sub-Saharan Africa [19]. Across 8 trials, 2 doses of SP reduced the incidence of LBW by 27% (95% confidence interval [CI], 13%–59%) [11]. A further meta-analysis showed that across 7 trials, the impact of ≥3 doses vs 2 doses significantly improved reductions in LBW by 20% (95% CI, 6%–31%) [10]. Given the absence of further placebo-controlled trial data following the introduction of IPTp-SP as policy across Africa, a meta-analysis of existing trial data and 50 observational studies that documented SP dosing and birthweight outcomes was performed, and study site data were matched to levels of SP resistance [20]. This updated analysis demonstrated an overall 21% (95% CI, 17%–25%) reduction in LBW associated with every additional dose of SP across all gravidities, but the impact declined with increasing SP resistance [20].

There has been growing concerns that increasing levels of mutations resistant to SP across Africa might reduce the impact of an otherwise simple intervention on LBW and other maternal health indicators [21–23]. Parasite resistance to SP results from successive acquisition of polymorphisms in the parasite genes that encode the targets of sulfadoxine and pyrimethamine: dihydropteroate synthase (*dhps*) and dihydrofolate reductase (*dhfr*), respectively. In areas with a high prevalence of quintuple mutant, SP has been shown to remain effective as IPTp [10, 20, 24]. However, the emergence of a more highly resistant sextuple mutant parasite was associated with a loss of IPTp-SP efficacy [20–22, 25, 26]. On the other hand, a pooled analysis of IPTp trials suggests that IPTp-SP may retain efficacy against LBW through a mechanism that is unrelated to malaria, as evidenced by similar efficacy across gravidity, whereas protection against malaria (as exemplified by the effect of dihydroartemisinin piperaquine) would have more impact for primigravida mothers vs multigravida mothers [27]. Here, we analyze birthweight data from a surveillance system on the Kenyan coast to estimate the impact of varying doses of SP in an area with high prevalence of molecular markers of SP resistance.

## METHODS

### Study Area and Context

Kilifi County is located on the Kenyan coast and is served by a maternity ward located at the referral hospital in Kilifi Township. Kilifi North and Kilifi South are 2 of 7 subcounties that constitute Kilifi County and are closest to the county hospital, within 45 km at their most distal reaches. The county has witnessed a sustained reduction in the intensity of malaria transmission since 2000 [28]. Since 2014, the annual prevalence of *P. falciparum* in children aged 2–10 years was on average 2.2% in Kilifi North and 9.2% in Kilifi South, respectively [29].

Using 2 presumptive doses of SP, IPTp was first introduced in Kilifi in 1996 during a placebo-controlled trial [30, 31] and later introduced as part of national reproductive health policy by the Ministry of Health in early 2000 [32]. In 2010, the national policy on IPTp using 2 SP doses was restricted to pregnant women living in moderate- to high-transmission areas along the Kenyan coast and in Western Kenya [33]. Two doses were also recommended in revisions to guidelines in 2012 [34] and 2014 [35]. Guidelines revised in 2016 recommended the administration of SP by direct observation with each scheduled visit after quickening to ensure women receive a minimum of 3 doses at 4-week intervals [36]. It is hard to ascertain which year these revised recommendations were implemented but a conservative estimate is 2015. These revisions were implemented in Coastal and Western Kenya, which are priority areas for the delivery of long-lasting insecticide-treated nets (LLINs) to pregnant women at ANC clinics. Since 2015, access to LLINs were augmented during catch-up mass campaigns in 2017 and 2020. On average, 72% of women aged 15–49 years who are residents within the Kilifi Health and Demographic Surveillance Area (which covers a large part of Kilifi North and Kilifi South subcounties [37]) sleep under an LLIN (unpublished data).

In Kilifi since 2015, more than 90% of sampled parasites have mutation substitutions for 437G and 540E on the *Pfdhps* and 108N, N54I, and 59R on the *Pfdhfr* [38]. A recent review examining the impact of resistance on the protective effect of SP on low birthweight deliveries regards high resistance as *dhps* substitutions where Lys540Glu ≥60% and Ala581Gly <10% and as very high where Lys540Glu ≥60% and Ala581Gly ≥10% where impact on LBW of IPTp with SP was no longer significant [20]. Frequencies of the 540E and the 581G *dhps* mutations are currently >90% and <2%, respectively, in Kilifi [38, 39].

### Maternity Ward Surveillance at Kilifi County Hospital

The Kilifi Perinatal and Maternity Research Study was established to standardize the maternal admissions procedure at Kilifi County Hospital and improve the standard of care [40]. The clinical surveillance was established in January 2011 in collaboration with hospital management and involves all hospital staff who provide maternity care. All mothers who present in labor were registered on admission by trained fieldworkers based in the maternity ward 24/7. Data were collected on residential addresses of all mothers, age, educational attainment, gravidity, and information recorded on the ANC card (human immunodeficiency virus [HIV] status, ANC visit, and SP frequency). Mid–upper arm circumference (MUAC) was recorded, and malnourished women were defined as mothers with an MUAC <23 cm [41, 42]. If an HIV test had not been performed as part of a routine ANC visit, a rapid test was offered to women at admission. There was, however, absence of reliable documentation of LLIN use by women at ANC visits and during delivery in the hospital. At delivery, information was recorded on newborn outcome, sex, gestational age determined by calculating either the difference between the date of delivery and date of the last menstruation period recorded on the ANC card or as determined by the midwife by measuring fundal height upon delivery of the child. Weight was measured within the first hour of birth using a balanced digital Seca scale. All data were captured using a customized tool built on a Hypertext Preprocessor (PHP) web-based interface, and data were saved onto the My Structured Query Language (MySQL) database.

### Statistical Analyses

Data were abstracted for 72 months from January 2015 to December 2021 to cover a period when national IPTp recommendations changed from 2 doses to at least 3 doses of SP in the second and third trimesters. Data were incomplete for 12 months due to health worker strikes in 2016, 2017, 2020, and 2021 [43, 44]. There remains a large access gap in the provision of SP to pregnant women (Supplementary Figure 1). In part, this gap has been a result of stock-outs of SP at clinics, most notably at the end of 2014 and the first half of 2016 when county financing led to procurement problems [45, 46]. Information on each mother’s residence was used to identify those who were resident in the 2 most proximal subcounties of Kilifi North and Kilifi South, restricting the analysis to those with the easiest access to the maternity ward. Descriptive statistics included proportions with 95% CIs and means with standard deviation (SD). A *χ*^2^ test was used to test the association between LBW and categorical variables.

**Figure 1.**
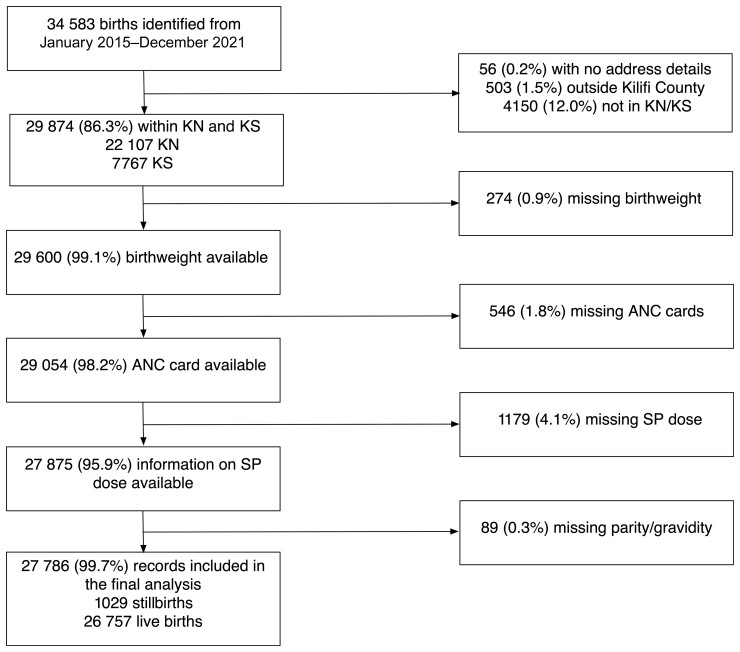
Inclusion criteria.

A logistic regression model was developed to estimate the effect of increasing SP doses on the risk of LBW deliveries (<2500 g), overall and stratified by gravidity status. Stillbirths were excluded from the analysis but presented separately. The models were adjusted for maternal age, marital status, maternal education (no formal education or primary, secondary, or higher education), residency (Kilifi North or Kilifi South), MUAC, preterm delivery (<37 weeks gestational), ANC utilization (1–2, 3–4, and ≥5 visits), HIV status, singleton or multiple births, newborn’s sex, season (dry or wet), and year of birth. Mothers who had missing information on ANC utilization, gravidity, SP doses, delivery outcome recorded as stillbirths, and newborn with missing birthweight were excluded from the LBW analysis (Figure 1). Variance inflation factors were examined for predictors included in the models to assess potential collinearity. A backward stepwise elimination technique was used to select the best fitting model with Hosmer and Lemeshow goodness-of-fit tests and area under the receiver operating characteristic curve used to assess model fit and assumptions. Adjusted odds ratios (aORs) with 95% CIs were defined. Data analyses were performed using STATA version 17.0 (Stata Corp, College Station, TX) and statistical software R version 4.1.0.

### Ethical Approval

The Kenya Medical Research Institute Scientific and Ethics Review Unit approved the study.

## RESULTS

Outcomes on 29 874 deliveries from Kilifi North and Kilifi South subcounties were recorded, representing 86.3% of all deliveries at the county hospital maternity ward between 2015 and 2021. Of these deliveries, 274 (0.9%) were excluded from the analysis because they had missing birthweight, 546 (1.8%) had missing information on ANC visits, 1179 (4.1%) had missing SP dose, and 89 (0.3%) had missing information on parity (Figure 1). Of the remaining 27 786 deliveries, 1029 were stillbirths, 1624 were multiple births (789 twins, 14 triplets, and 1 quadruplet), and 3540 (13.1%) were pre-term as assessed by estimated gestational age. Most mothers had attended an ANC clinic (99.7%) at least once, with 82.9% having 3 or more ANC follow-up visits (Table 1). ANC documentation of SP doses ranged from zero (13.7%) to 7 doses (0.02%); for 13 999 (51.9%) births, the mothers had received ≥3 doses (Table 1).

**Table 1.** Characteristic of Mothers and Newborns Delivered at Kilifi County Hospital

Characteristic Mothers	Summary Statistics n = 26 961	Characteristic Newborns	Summary Statistics n = 27 786
			
Antenatal care visits, n (%)		Mean weight (SD), g	2943.9 (598.8)
0	77 (0.3)	Low birthweight, n (%)	4875 (17.5)
1–2	4538 (16.8)	Mean gestation age (SD), weeks	39.0 (2.8)
3–4	12 644 (46.9)	Preterm, n (%)	3540 (13.1)
5+	9702 (36.0)	Birth state, n (%)	
Sulfadoxine–pyrimethamine dose, n (%)		Stillbirth	1029 (3.7)
0	3676 (13.6)	Live birth	26 757 (96.3)
1	4046 (15.0)	Number of births, n (%)	
2	5240 (19.4)	Singleton	26 162 (94.2)
3	6144 (22.8)	Multiples	1624 (5.8)
4	5345 (19.8)	Female, n (%)	13 352 (48.1)
5+	2510 (9.3)	Year of birth, n (%)	
Primigravida, n (%)	10 339 (38.4)	2015	4093 (14.7)
Mean maternal age (SD), years	26.4 (6.3)	2016	3699 (13.3)
Human immunodeficiency virus positive, n (%)	1014 (3.9)	2017	1176 (4.2)
Mean mid–upper arm circumference (SD), cm	26.3 (3.6)	2018	5137 (18.5)
Education level, n (%)		2019	5320 (19.2)
None	2407 (9)	2020	3657 (13.2)
Primary	15 241 (56.9)	2021	4704 (16.9)
Secondary	6000 (22.4)		
Higher	3153 (11.8)		
Marital status, n (%)			
Married	24385 (90.8)		
Single	2315 (8.6)		
Divorced	128 (0.5)		
Widowed	40 (0.2)		
Residency, n (%)			
Kilifi North	20 031 (74.3)		
Kilifi South	6930 (25.7)		
Wet season, n (%)	12 645 (46.9)		

Categorical variables are expressed as n (%) and continuous variables are expressed as mean (SD).

Abbreviation: SD, standard deviation.

### Impact of SP Dose on LBW

The mean birthweight was 2973 g (±559), and the prevalence of LBW was 16.0% (95% CI, 15.5%–16.4%) among livebirths. The prevalence of LBW was significantly higher in primigravida mothers (18.0%) than multigravida mothers (14.8%; *P* < .001; Supplementary Table 1). The prevalence of LBW among mothers who received at least 1 dose of IPTp-SP was 15.3% (3546 of 23 146) vs 20.1% (727 of 3611) among those who did not take any dose of IPTp-SP (*P* < .001). An increase in IPTp-SP dosage was associated with an increased reduction in the risk of LBW. This dose-associated impact was statistically significant overall and by gravidity in univariable and multivariable regression (Table 2). The multivariable regression models across all gravidities and stratified by gravidity were adjusted for factors listed in the footnote of Table 2. Overall, IPTp-SP was associated with a significant reduction in LBW of 13% (aOR, 0.87; 95% CI, .76–.99; *P* < .001) in the 2-dose group compared with the no-dose group. Three or more doses were associated with an even lower risk of LBW (aOR, 0.73; 95% CI, .64–.83; *P* < .001) compared with no doses. There was a dose-scaling effect, with the lowest risk of LBW seen in the 5 or more SP-dose group (aOR, 0.54; 95% CI, .44–.67; Table 2) compared with the no-dose group. In the primigravida group, ≥3 SP doses also showed a protective effect (aOR, 0.78; 95% CI, .63–.97; *P* = .0147) compared with no doses, although the effect was mostly evident in the 4 and 5 or more SP-dose group (Table 2). Among multigravida women, there was evidence of a dose-scaling effect, with ≥3 SP doses being associated with a 30% reduction (aOR, 0.70; 95% CI, .60–.82, *P* < .001) in the risk of LBW (Table 2) compared with no doses. There was no evidence that the impact of SP varied over time (Supplementary Figure 2) or that the impact of SP varied in the low-transmission north vs the higher-transmission south of Kilifi County (Supplementary Figure 3).

**Table 2.** Effect of Sulfadoxine–Pyrimethamine Dose on Risk of Low Birthweight Deliveries: Overall and by Parity/Gravida Status

Low Birthweight Deliveries	No. (%)	Crude OR (95% CI)	*P* Value	Adjusted OR (95% CI)	*P* Value
Overall		n = 26 757		n = 24 479	
SP dose					
0	727/3611 (20.1)	1.00	<.001	1.00	<.001
1	836/3972 (21.1)	1.06 (.95–1.18)		.92 (.80–1.06)	
2	957/5184 (18.5)	.90 (.81–1.00)		.87 (.76–.99)	
3	899/6097 (14.7)	.69 (.62–.76)		.80 (.70–.93)	
4	645/5382 (12.0)	.54 (.48–.61)		.69 (.59–.80)	
5+	209/2511 (8.3)	.36 (.31–.42)		.54 (.44–.67)	
Primigravida		n = 10 214		n = 9444	
SP dose					
0	249/1184 (21.0)	1.00	<.001	1.00	.0024
1	347/1407 (24.7)	1.23 (1.02–1.48)		1.01 (.80–1.27)	
2	439/1977 (22.2)	1.07 (.90–1.28)		.97 (.78–1.21)	
3	389/2318 (16.8)	.76 (.63–.90)		.87 (.69–1.09)	
4	304/2240 (13.6)	.59 (.49–.71)		.74 (.58–.94)	
5+	105/1088 (9.7)	.40 (.31–.51)		.59 (.43–.81)	
Multigravida		n = 16 543		n = 15 158	
SP dose					
0	478/2427 (19.7)	1.00	<.001	1.00	<.001
1	489/2565 (19.1)	.96 (.83–1.11)		.84 (.71–1.01)	
2	518/3207 (16.2)	.79 (.68–.90)		.80 (.68–.95)	
3	510/3779 (13.5)	.64 (.55–.73)		.77 (.65–.92)	
4	341/3142 (10.9)	.50 (.43–.58)		.67 (.55–.80)	
5+	104/1423 (7.3)	.32 (.26–.40)		53 (.40–.69)	

The overall model was adjusted for mother’s mean mid–upper arm circumference (MUAC), marital status, education level, gravidity, antenatal care (ANC) visits, residency (Kilifi North vs Kilifi South), year of birth, gestational age, multiple births, and sex of the newborn(s). The model for primigravids was adjusted only for mother’s age, mother’s MUAC, education level, ANC visits, year of birth, season, gestational age, multiple births, and sex of the newborn(s). The model for multigravidas was adjusted only for mother’s MUAC, ANC visits, residency (Kilifi North vs Kilifi South), gestational age, multiple births, and sex of the newborn(s).

Abbreviations: CI, confidence interval; OR, odds ratio; SP, sulfadoxine–pyrimethamine.

### Impact of SP Dose on Stillbirths

Overall, 3 or more doses of IPTp-SP was also associated with a 21% reduction in stillbirth deliveries (aOR, 0.79; 95% CI, .65–.97; *P* = .0438) compared with no doses. However, due to the low numbers of stillbirths, there was no clear evidence of a dose-scaling effect (Table 3). Similarly, the aOR was 0.66 (95% CI, .46–.92; *P* = .0144) among primigravida mothers who received 3 or more doses compared with those who received no doses. However, among multigravida mothers, there was no evidence that 3 or more doses of IPTp-SP reduced the risk of stillbirths (aOR, 0.83; 95% CI, .64–1.08; *P* = .2434) compared with no doses.

**Table 3.** Effect of Sulfadoxine–Pyrimethamine Dose on Risk of Stillbirths: Overall and by Parity/Gravida Status

Stillbirths	No. (%)	Crude OR (95% CI)	*P* Value	Adjusted OR (95% CI)	*P* Value
Overall		n = 27 786		n = 26 792	
SP dose					
0	192/3803 (5.1)	1.00	<.001	1.00	.0037
1	228/4200 (5.4)	1.08 (.89–1.31)		1.03 (.83–1.27)	
2	225/5409 (4.2)	.82 (.67–.99)		.89 (.72–1.10)	
3	221/6318 (3.5)	.68 (.56–.83)		.93 (.74–1.15)	
4	113/5495 (2.1)	.39 (.31–.50)		.63 (.49–.82)	
5+	50/2561 (1.9)	.37 (.27–.51)		.72 (.51–1.01)	
Primigravida		n = 10 573		n = 10 263	
SP dose					
0	59/1243 (4.7)	1.00	.0129	1.00	.0218
1	70/1477 (4.7)	1.00 (.70–1.42)		.99 (.68–1.45)	
2	81/2058 (3.9)	.82 (.58–1.16)		.90 (.62–1.29)	
3	77/2395 (3.2)	.67 (.47–.94)		.78 (.54–1.12)	
4	48/2288 (2.1)	.43 (.29–.63)		.56 (.38–.84)	
5+	24/1112 (2.2)	.44 (.27–.72)		.60 (.36–.98)	
Multigravida		n = 17 213		n = 15 761	
SP dose					
0	133/2560 (5.2)	1.00	<.001	1.00	.0272
1	158/2723 (5.8)	1.12 (.89–1.42)		1.06 (.80–1.40)	
2	144/3351 (4.3)	.82 (.64–1.04)		.88 (.67–1.16)	
3	144/3923 (3.7)	.70 (.55–.88)		1.00 (.75–1.33)	
4	65/3207 (2.0)	.38 (.28–.51)		.63 (.45–.88)	
5+	26/1449 (1.8%)	.33 (.22–.51)		.70 (.44–1.13)	

The overall model was adjusted for mother’s age, education level, parity/gravida status, antenatal care (ANC) visits, residency (Kilifi North vs Kilifi South), and gestational age. The model for primigravids was adjusted only for mother’s age, education level, and gestational age. The model for multigravidas was adjusted only for mother’s mean mid–upper arm circumference, ANC visits, residency (Kilifi North vs Kilifi South). and gestational age.

Abbreviations: CI, confidence interval; OR, odds ratio; SP, sulfadoxine–pyrimethamine.

## DISCUSSION

The intensity of malaria transmission in Kilifi County has declined dramatically over the last 20 years, and childhood infection prevalence is now only 2.4% [29]. However, new infections are increasingly likely to include those with high frequencies of *dhps* and *dhfr* mutations that reduce the efficacy of SP [38]; the quintuple resistance mutation (including *dhps*-K540E) is >90% [38, 39]. Despite a low probability of infection but a high probability of encountering an SP-resistant infection, the use of SP as IPTp continues to be associated with a reduction in the risk of LBW deliveries (27%; 95% CI, 17%–36%) across all gravidities. Importantly, protection increased with increasing doses of SP prior to delivery (Table 2). Equally, protection is provided against stillbirths (21%; 95% CI, 3%–35%) overall and in primigravida women.

Observational studies are always subject to “adherer effects” [47]. Those who use services have a different, broader health awareness or improved health status compared with those who do not access services. These effects may have influenced previous meta-analyses of observational studies, and the heterogeneity between studies may reflect differential biases rather than true effects [20, 26]. Here, we used longitudinal data during a period of increasing resistance where we rigorously adjusted for a range of possible confounders including previous experience (parity), maternal age, nutrition, educational status, gestational age, and ANC clinic attendance that would limit opportunities for multiple SP doses. Health worker strikes and ANC clinic SP stock-outs provided a natural experiment that provided system-level, rather than pregnant women individual-level, failures to limit SP use during the surveillance interval. Furthermore, when the analysis was restricted to women who attended an ANC clinic at least 3 times, that is, women with defined high service use and having a maximal opportunity for at least 3 doses, we observed similar impacts and dose response in the overall analysis (Supplementary Table 2).

Our findings of sustained impact on birth outcomes with SP in areas with high levels of SP resistance mutations are consistent with those from previous reports [10, 20, 24, 26, 48]. Although the quintuple resistance mutation is high on the Kenyan coast, pregnant women in this area still benefit from receiving SP. It is conceivable that SP offers broad-spectrum antimicrobial protection that impacts the biome of the maternal–fetus environment in utero, promoting maternal weight gain [26, 27, 49, 50] and, therefore, is independent of SP’s malaria activity. In addition, SP may not be entirely ineffectual in protecting a mother from a small number of new, incident, sensitive infections that bind to the placenta or in stimulating maternal immunity [4, 26]. The consistent impact size across gravidity, across a temporal trend of increasing SP resistance [38] among falling malaria transmission, and across regions of differing malaria transmission [51] favors the non-malaria hypothesis. However, the biological mechanism of action remains unclear and requires further investigation.

ANC clinic attendance among hospital deliveries was high; 83% of women had attended an ANC clinic during the pregnancy at least 3 times (Table 1). Sample household surveys, undertaken as part of national malaria, health, and demographic surveys in 2014, 2015, and 2020, taken by 332 women who had been pregnant in the 24 months prior to the survey in Kilifi County, of whom 88% had attended an ANC clinic ≥3 times; however, only 32% reported taking ≥3 SP doses [52–54]. There remains a large access gap in the provision of SP to pregnant women. One caveat is that we were unable to examine the relative contributions of SP vs LLIN or their combination. LLIN coverage among women of reproductive age in our study area was high at >70%, and our results of the impact of increasing SP doses on LBW should be viewed in this context. Additionally, although multivariable models may have reduced the potential for bias, residual confounding cannot be excluded. Last, this study was conducted at a single site of low malaria transmission and high SP resistance; therefore, the results may not be generalizable to high-transmission settings.

Surveillance at maternity wards offers a unique opportunity to continuously monitor the effectiveness of IPTp against important health outcomes [55]. Molecular surveillance of resistance mutations, while providing important contextual information, should be used together with health outcome data in order to guide policy. Surveillance of routine hospital deliveries would provide useful information on the impact of SP across a range of malaria transmission intensities and parasite-resistance landscapes.

Our results demonstrate a high level of dose-dependent protection of SP provided at ANC clinics against LBW deliveries and stillbirths despite a high prevalence of *Pfdhps*-K540E mutations. These results suggest that in areas experiencing high SP resistance, IPTp-SP should continue to be used and that when the mechanisms of action are better understood, IPTp-SP might have some value in areas of very low parasite transmission.

## Supplementary Data

Supplementary materials are available at *Clinical Infectious Diseases* online. Consisting of data provided by the authors to benefit the reader, the posted materials are not copyedited and are the sole responsibility of the authors, so questions or comments should be addressed to the corresponding author.

## Notes

***Author contributions*.** R. W. S. and A. K. conceived of the study. A. K. analyzed and interpreted the data and drafted the manuscript, and R. W. S., P. B., and J. A. B. provided input. M. M. and D. A. assisted with data extraction, cleaning, and analysis. A. C. S., A. N., and S. M. coordinated the data collection process at Kilifi County Hospital. J. A. B. and A. C. S. acquired funding and were involved in project administration at Kilifi County Hospital. All authors reviewed and approved the final manuscript as submitted.

***Acknowledgments.*** The authors are grateful to the Kilifi Perinatal and Maternal Research Group and the fieldworker team. The authors also appreciate all the staff and patients at Kilifi County Hospital. They also acknowledge Lynette Isabella Ochola-Oyier and Irene Omedo for information on sulfadoxine–pyrimethamine resistance and Anitah Cherono for help with national household survey data. All authors are grateful for the support from the Wellcome Trust to the Core Award for the East Africa Major Overseas Programme.

***Data sharing.*** Data that support the findings of this study are available from the Kenya Medical Research Institute (KEMRI) Institutional Data Access/Ethics Committee. Details of the guideline can be found in the KEMRI-Wellcome data sharing guidelines (https://kemri-wellcome.org/about-us/#ChildVerticalTab_15). Access to data is provided via the KEMRI Wellcome Data Governance Committee (dgc@kemri-wellcome.org).

***Disclaimer.*** The funders had no role in study design, data collection and analysis, preparation of the manuscript, or decision to publish. This study is published with the permission of the director of the KEMRI.

***Financial support*.** This work was supported by the Wellcome Trust as part of the Principal Research Fellowship (212176/Z/18/Z) to R. W. S., which also provided support to A. K. and M. M. Medical Research Council (MRC)/UK Department for International Development (DFID)/Wellcome Trust (MR/M007367/1) and the Bill and Melinda Gates Foundation (OPP1131320) supported J. A. B. The Wellcome Trust also provided supported to A. C. S. as part of the Career Development Fellowship (205184/Z/16/Z).

## Supplementary Material

ciac509_Supplementary_Data
