# Supplementary material for: Impact of Intermittent Presumptive Treatment for Malaria in Pregnancy on Hospital Birth Outcomes on the Kenyan Coast
Source: Clin Infect Dis. Author manuscript; Available in PMC 2023 Feb 15. (PMC9907553; doi:10.1093/cid/ciac509)
Supplement: Supplementary Data [file EMS153586-supplement-Supplementary_Data.docx]

**Supplementary Table 1: Univariable and multivariable logistic regression results on the risk of LBW across all gravidas**

| **LBW** | **Crude OR**  **(95% CI)** | **P value** | **Adjusted OR**  **(95% CI)** | **P value** |
| --- | --- | --- | --- | --- |
| SP dose |  |  |  |  |
| 0 | 1.00 | <0.001 | 1.00 | <0.001 |
| 1 | 1.06 (0.95, 1.18) |  | 0.92 (0.80, 1.06) |  |
| 2 | 0.90 (0.81, 1.00) |  | 0.87 (0.76, 0.99) |  |
| 3 | 0.69 (0.62, 0.76) |  | 0.80 (0.70, 0.93) |  |
| 4 | 0.54 (0.48, 0.61) |  | 0.69 (0.59, 0.80) |  |
| 5 + | 0.36 (0.31, 0.42) |  | 0.54 (0.44, 0.67) |  |
| ANC visits |  |  |  |  |
| 0 visits | 1.00 | <0.001 | 1.00 | <0.001 |
| 1-2 visits | 0.35 (0.22, 0.57) |  | 0.57 (0.29, 1.16) |  |
| 3-4 visits | 0.21 (0.13, 0.33) |  | 0.40 (0.20, 0.81) |  |
| 5+ visits | 0.13 (0.08, 0.22) |  | 0.32 (0.16, 0.66) |  |
| Gradivity |  |  |  |  |
| Primigravida | 1.00 | <0.001 | 1.00 | <0.001 |
| Multigravida | 0.79 (0.74, 0.85) |  | 0.59 (0.54, 0.64) |  |
| MUAC |  |  |  |  |
| < 23cm | 1.00 | <0.001 | 1.00 | <0.001 |
| ≥ 23cm | 0.65 (0.59, 0.71) |  | 0.75 (0.67, 0.83) |  |
| Preterm |  |  |  |  |
| < 37 weeks | 1.00 | <0.001 | 1.00 | <0.001 |
| ≥ 37 weeks | 0.15 (0.14, 0.17) |  | 0.18 (0.16, 0.20) |  |
| Number of births | |  |  |  |
| Singleton | 1.00 | <0.001 | 1.00 | <0.001 |
| Multiples | 13.97 (12.48, 15.64) |  | 14.22 (12.50, 16.18) |  |
| Year of birth |  |  |  |  |
| 2015 | 1.00 | 0.105 | 1.00 | 0.0018 |
| 2016 | 0.99 (0.88, 1.12) |  | 1.01 (0.86, 1.17) |  |
| 2017 | 0.95 (0.79, 1.14) |  | 0.80 (0.63, 1.02) |  |
| 2018 | 0.94 (0.84, 1.05) |  | 1.13 (0.98, 1.31) |  |
| 2019 | 0.87 (0.78, 0.97) |  | 1.07 (0.93, 1.24) |  |
| 2020 | 0.98 (0.87, 1.11) |  | 1.26 (1.08, 1.47) |  |
| 2021 | 1.02 (0.91, 1.14) |  | 1.04 (0.90, 1.20) |  |
| Marital status |  |  |  |  |
| Married | 1.00 | 0.016 | 1.00 | 0.048 |
| Single | 1.15 (1.02, 1.28) |  | 0.87 (0.75, 1.00) |  |
| Divorced | 1.39 (0.90, 2.14) |  | 1.23 (0.74, 2.06) |  |
| Widowed | 0.30 (0.07, 1.23) |  | 0.26 (0.06, 1.14) |  |
| Education level |  |  |  |  |
| None | 1.00 | <0.001 | 1.00 | <0.001 |
| Primary | 0.95 (0.85, 1.07) |  | 0.98 (0.85, 1.13) |  |
| Seconday | 0.77 (0.68, 0.87) |  | 0.81 (0.69, 0.95) |  |
| Higher | 0.63 (0.54, 0.73) |  | 0.77 (0.64, 0.93) |  |
| Sub counties |  |  |  |  |
| Kilifi North | 1.00 | <0.001 | 1.00 | <0.001 |
| Kilifi South | 1.40 (1.30, 1.50) |  | 1.19 (1.09, 1.29) |  |
| Newborn sex |  |  |  |  |
| Female | 1.00 | <0.001 | 1.00 | <0.001 |
| Male | 0.75 (0.71, 0.80) |  | 0.69 (0.64, 0.75) |  |

**Supplementary Table 2: Effect of SP dose on the risk of LBW deliveries, overall across all gravidas among women who attended ANC ≥3 times**

| **LBW** | **No. (%)** | **Crude OR (95% CI)** | **P value** | **Adjusted OR (95% CI)** | **P value** |
| --- | --- | --- | --- | --- | --- |
| **Overall** |  | **n=22,311** |  | **n=20,554** |  |
| SP dose |  |  |  |  |  |
| 0 | 2,707 (12.1%) | 1.00 | <0.001 | 1.00 | <0.001 |
| 1 | 2,234 (10.0%) | 1.01 (0.87, 1.17) |  | 0.89 (0.74, 1.08) |  |
| 2 | 3,492 (15.7%) | 1.01 (0.88, 1.15) |  | 0.96 (0.81, 1.14) |  |
| 3 | 6,017 (27.0%) | 0.85 (0.75, 0.96) |  | 0.83 (0.71, 0.97) |  |
| 4 | 5,355 (24.0%) | 0.67 (0.59, 0.77) |  | 0.70 (0.60, 0.83) |  |
| 5+ | 2,506 (11.2%) | 0.45 (0.38, 0.54) |  | 0.55 (0.44, 0.69) |  |

The overall model was adjusted for mothers MUAC, marital status, education level, parity/gravida status, ANC visits, residency (Kilifi North vs Kilifi South), year of birth, gestation age, multiple births and sex of the new-borns.

**Supplementary Figure 1: The distribution of SP dose over time**

**
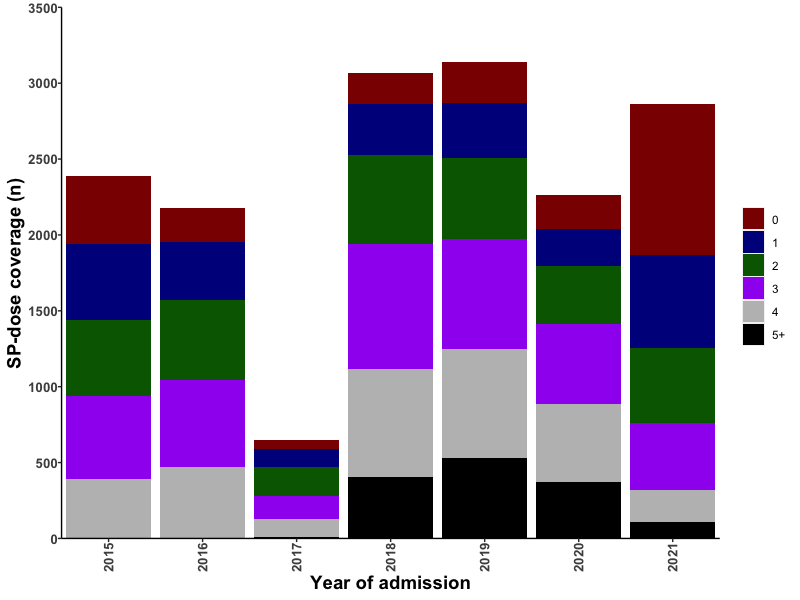
**

**Supplementary Figure 2: The interaction between the effect of ≥3 SP dose compared to <3 SP doses and year on the risk of LBW deliveries across all gravidas**

SP dose was categorized as < 3 doses vs ≥ 3 doses.The model was adjusted for mothers MUAC, marital status, education level, gravidity, ANC visits, residency, year of birth, gestation age, multiple births, sex of the new-borns, SP dose and the interaction between SP dose and year of birth. These are the results of the interaction terms between SP dose and year with 2015 are the reference year. The interaction was non-significant (p= 0.101) hence the effect of SP doses on LBW did not appear to be modified over time.

**Supplementary Figure 3: Effect of SP dose on the risk of LBW deliveries in Kilifi North and Kilifi South**

 The model was adjusted for mothers MUAC, education level, gravidity, ANC visits, year of birth, gestation age, multiple births and sex of the new-borns.
